# Burstein-Moss Effect Behind Au Surface Plasmon Enhanced Intrinsic Emission of ZnO Microdisks

**DOI:** 10.1038/srep36194

**Published:** 2016-11-02

**Authors:** Qiuxiang Zhu, Junfeng Lu, Yueyue Wang, Feifei Qin, Zengliang Shi, Chunxiang Xu

**Affiliations:** 1 State Key Laboratory of Bioelectronics, School of Biological Science & Medical Engineering, Southeast University, Nanjing 210096, China; 2 College of Communication and Electronic Engineering, Hunan City University, Yiyang 413000, China

## Abstract

In this paper, ZnO microdisks with sputtering of Au nanoparticles were prepared to explore their plasmon/exciton coupling effect. An obvious blue shift and enhanced excitonic emission intensity were observed in the PL spectra of as-grown and Au-sputtered ZnO samples at room temperature. The investigation on the absorption spectra and temperature-dependent PL spectra has been demonstrated the Burstein-Moss effect behind the optical phenomena. These results revealed the coupling dynamics between the metal localized surface plasmon and semiconductor exciton.

Improved luminescent efficiency and the brightness are everlasting topics for fundamental research and industrial application of semiconductor light-emitting devices. As one of the most promising candidate materials for short-wavelength optoelectronic devices, zinc oxide (ZnO) has been paid a considerable attention on its ultraviolet (UV) spontaneous and simulated emission based on the wide direct band gap (3.37 eV) and large exciton binding energy (60 meV) at room temperature^1^,^2^. In the past decades, surface plasmons (SPs) mediated emissions have been attracted extensive research interests as an effective way to improve the band edge emission of ZnO. For examples, Wang *et al*.^3^ demonstrated 11-fold enhancement of lasing by sputtering of Au nanoparticles (NPs) on the surface of ZnO nanotubes. Also, Xiao *et al*.^4^ reported 4-fold near-band emission enhancement by imbedding of Ag NPs in the ZnO film. In addition, Lin *et al*.^5^ found that the bandgap emission of the ZnO nanorods can be greatly enhanced by employing SPs of Pt NPs. Lu *et al*.^6^ also showed more than 170-fold enhancement of the band edge emission of ZnO microrods by decorating Al NPs. These enhancements are employed metal NPs on ZnO based on the surface plasmon resonance effect between SPs of metal NPs and ZnO excitons. However, the mechanism metal/semiconductor interaction underlying a plenty of optical phenomena is not yet clear, besides being generally attributed to SPs coupling.

On the other hand, the Fermi level is possibly raised over the conduction band of a semiconductor due to free electrons filling for band renormalization in a heavily doped case. That is so-called Burstein-Moss (BM) effect, which would result in blue shift of the optical band gap^7^,^8^,^9^. The BM effect is not a blue shift of the intrinsic band gap of a semiconductor but a blue shift of the optical band gap as a consequence of the state-filling in the conduction band. Also, it can be well known that the magnitude of BM shift (∆_BM_) from free-electron theory is proportional to *n*_*e*_^2/3^, where *n*_*e*_ is the electron carrier concentration^10^,^11^. For examples, BM effect induced blue shift of the optical band gap has been observed in the Al-doped ZnO films^12^ and nanowires (NWs)^13^, Si and Ge-doped GaN^14^, and Re-doped MoS_2_ NPs^15^. Recently, Liu *et al*.^9^ demonstrated that the lasing wavelength of a single CdS NW laser significantly represented a blue shift of more than 20 nm at room temperature through the SP-enhanced BM effect. However, the coupling mechanism between SPs and semiconductor has no profound understanding, which is of importance to design the corresponding functional devices.

In this paper, the wurtzite structured ZnO microdisks were synthesized by a typical vapor-phase transport (VPT) method. The systematic room temperature and temperature-dependent photoluminescence (PL) properties of the as-grown and Au-sputtered ZnO samples were investigated to explore the coupling process between SPs of Au NPs and ZnO excitons. The theoretical simulation on the electrons filling and the resulting band renormalization were just matched with the experimental observation of the spectral shift, and further revealed the BM effect. The BM effect related inter-band emission enhancement and spectral blue shift were controlled facilely by adjusting the sputtering time. The PL dynamics validated that the BM effect in the ZnO/Au was enhanced initially and then decreased as a consequence of the hot electrons filling in the conduction band of ZnO by varying the temperature from 10 K to 300 K.

## Results and Discussion

**Figure 1:**
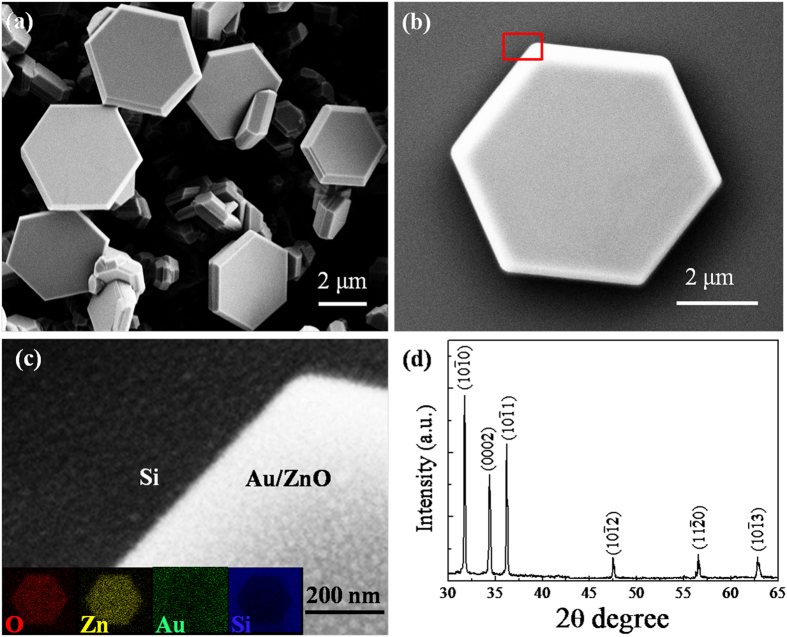
(**a**) SEM image of as-grown ZnO microdisks. (**b**) The enlarged SEM image of an individual ZnO microdisk. (**c**) The enlarged part taken from the red marked rectangle spot of (**b**), inserted with the element mapping of Zn, O, Au and Si corresponding to (**b**) after the decoration of Au NPs. (**d**) The XRD patterns of ZnO microdisks.

The SEM image of the produced ZnO sample in Fig. 1(a) clearly displays the hexagonal microdisks morphology. The enlarged SEM image of an individual ZnO microdisk in Fig. 1(b) further demonstrates the perfect hexagonal shape with smooth surface. Also, the enlarged part of the individual ZnO microdisk taken from the red rectangular region of Fig. 1(b) is showed in Fig. 1(c). From the figure, it can be noticed that the similar size and distribution of Au NPs sputtered at the same time can be observed on the surfaces of the ZnO and Si substrates. The inset in Fig. 1(c) further shows the element mapping images of the individual ZnO microdisk decorated with Au NPs. It clearly illustrates the uniform distribution of Zn, O, and Au elements in the ZnO/Au microdisk on Si substrate. The XRD pattern of the ZnO microdisks in Fig. 1(d) represents the six typical diffraction peaks from (100), (002), (101), (102), (110), (103) planes which correspond to the angles of 31.7°, 34.4°, 36.2°, 47.5°, 56.5°, and 62.9° respectively, and consistent with our previous reports on ZnO microdisks^16^,^17^. As indexed in the Figure, all the diffraction peaks are well matched with the wurtzite structured ZnO with lattice constants of *α* = 3.250Å and *c* = 5.207Å.

**Figure 2:**
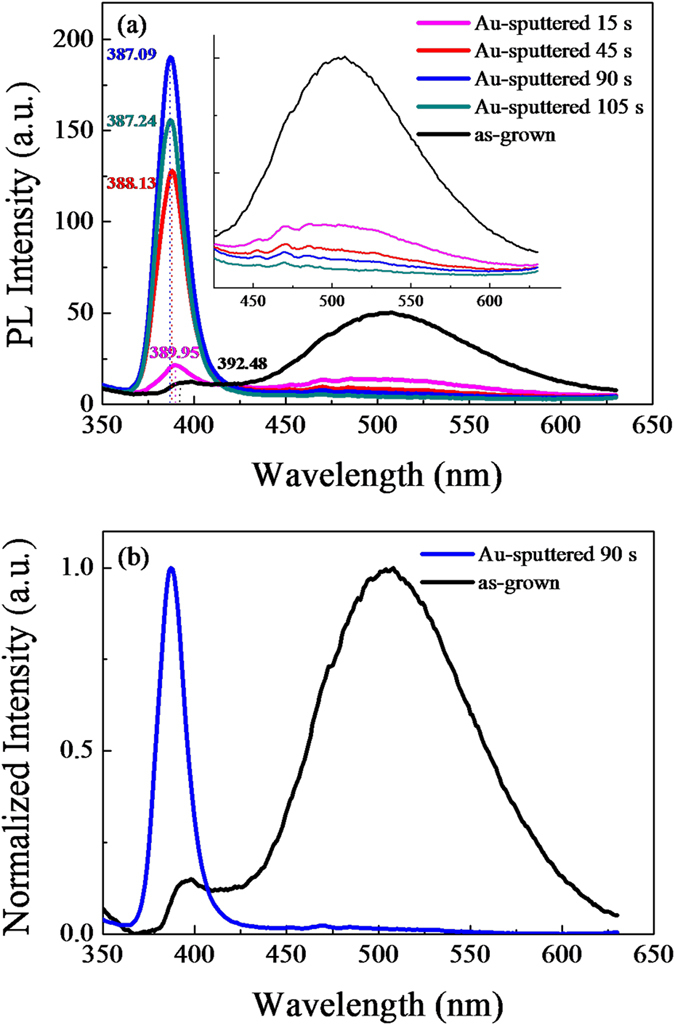
(**a**) Room temperature PL spectra of the ZnO microdisks before and after the sputtering of Au NPs. The inset spectra exhibit the magnified defect-related emission of all the samples. (**b**) The normalized PL spectra of the as-grown and Au-sputtered 90s samples.

The PL spectra of the ZnO microdisks were measured at room temperature before and after the sputtering of Au NPs. The as-grown ZnO microdisks exhibit a weak near band-gap emission (NBE) at around 392.48 nm and a strong broad defect-related emission band centered at 508.12 nm, as shown in Fig. 2. The intensity and their ratio of the NBE to the defect emission vary obviously with different sputtering time at the same excitation condition. For the ZnO sample sputtered for 15 s, the NBE at 389.95 nm increases a little while the defect peak intensity reduces near to the same level. It is found that the NBE enhances gradually while the defect emission reduces as the sputtering time is prolonged. For the sample sputtered for 45 s, the NBE at 388.13 nm is much stronger than the defect emission peak. As the sputtering time is extended to 90 s, the NBE at 387.09 nm reaches the maximum while the defect emission disappears almost. The strongest NBE of the Au-decorated sample is enhanced about 20 folds and blue shifted 5.39 nm (44 meV) relative to that of the as-grown ZnO microdisks. The normalized PL spectra in Fig. 2(b) demonstrated the spectral variation before and after Au decoration more clearly. When the sputtering time is extended to 105 s, the PL intensity decreases and the NBE red shifts a little (1.4 meV) to 387.24 nm relative to the maximum shifted one (sputtered for 90 s) but it still displays stronger UV emission and blue shift (42.6 meV) relative to the as-grown sample. In a word, the NBE emission enhances gradually to saturation and then reduces with blue shift, while the defect emission decreases sustainedly with prolongation of the sputtering time, as shown the inserted spectra in Fig. 2(a) more clearly. This indicates the influence of the size and spatial distribution of Au NPs on the PL behaviors of the ZnO microdisks.

**Figure 3:**
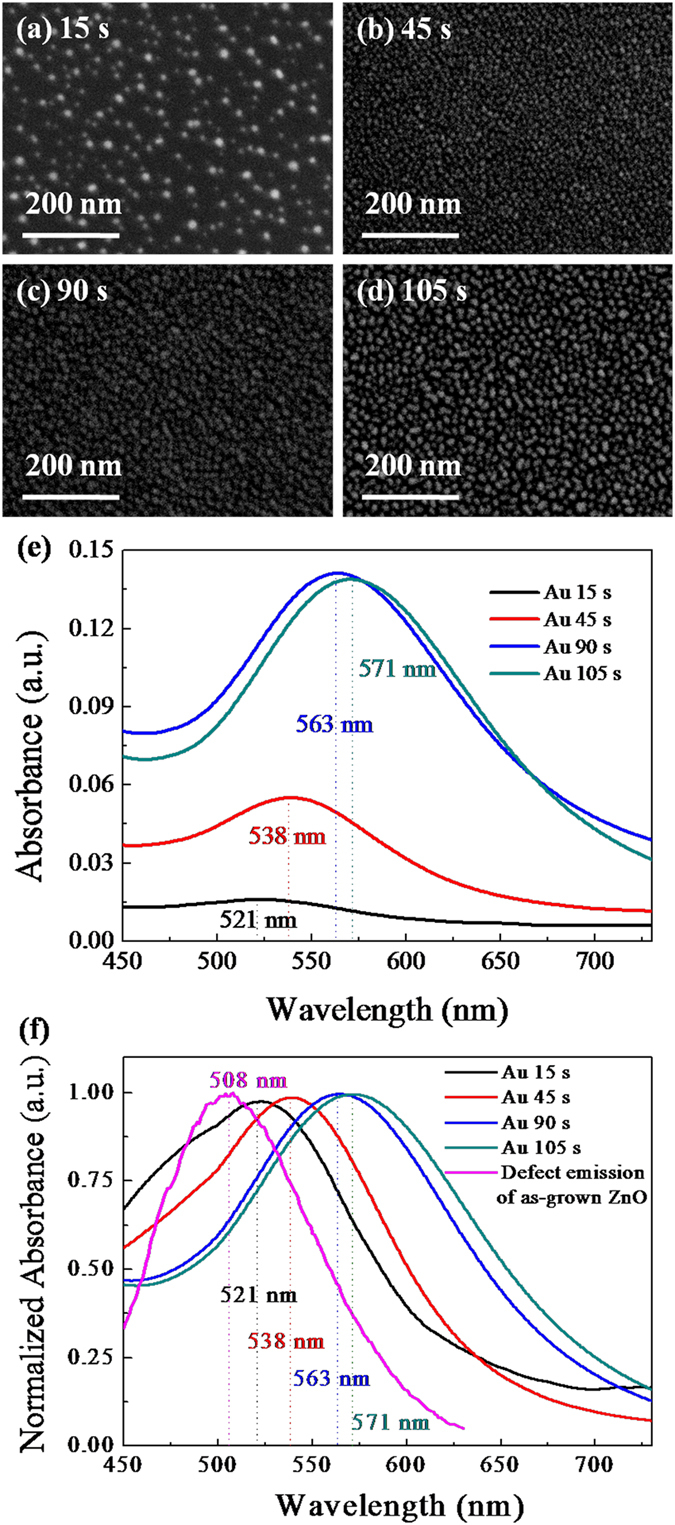
SEM images of Au NPs with different sputtering times (**a**) 15 s, (**b**) 45 s, (**c**) 90 s and (**d**) 105 s. (**e**) Absorption spectra of Au NPs with different sputtering times on the quartz substrates and (**f**) Normalized absorbance of Au NPs and the normalized defect-related emission of ZnO.

To understand the action of the Au NPs and the ZnO/Au interaction, the Au NPs were also collected on Si and quartz substrates under the same preparation conditions to character their morphologies and the corresponding optical behaviors, respectively. As shown the SEM images in Fig. 3(a–d), the Au NPs on Si substrates become bigger and denser with increasing of the sputtering time. The average size of NPs increases from 8 nm to 15 nm and 18 nm as the sputtering time increases from 15 s to 45 s and 90 s, respectively. When the sputtering time is increased to 105 s, the Au NPs are even merged together. The absorption spectra of Au NPs with different sputtering time on the clean quartz substrates are shown in Fig. 3(e). The absorption peaks locate at 521 nm, 538 nm, 563 nm, and 571 nm corresponding to the sputtering time of 15 s, 45 s, 90 s and 105 s, respectively. The absorbance of Au NPs is gradually increased when the sputtering is from 15 s to 90 s and then decreased a little with the sputtering time further increased to 105 s, which is consistent with the NBE intensity variation trend of ZnO microdisks. As the sputtering time is 90 s, the absorbance reaches the maximum corresponding to the strongest NBE intensity of ZnO/Au microdisks. Figure 3(f) displays the normalized absorption spectra of Au NPs with different sputtering time and the normalized spectrum of the defect-related emission for ZnO is also plotted for comparison. The spectral overlap and the absorption intensity make a joint effect on possible coupling between the Au SPs and the defect emission of ZnO. In the sputtering time of 15 s, the small Au NPs with average size of 8 nm distribute sparsely onto substrate and present weak absorption at 521 nm, which corresponds to a slight enhancement in NBE intensity of ZnO microdisks accompanying with the obvious decrease of the defect emission. As the size of Au NPs increases to 15 nm and 18 nm for the sputtering time of 45 s to 90 s, the spatial distribution becomes denser and the Au NPs have stronger absorption at 538 nm and the strongest absorption at 563 nm. In these cases, the NBE of ZnO microdisks can be enhanced gradually and then reached to the maximum while the defect emission disappeared almost. When the sputtering time is increased to 105 s, the Au NPs are merged together, as shown in Fig. 3(d), which induces a slight weaker absorption compared to the 90 s sample. The absorption peak can be further shifted to 571 nm, which corresponds to a slight decrease of the NBE due to an increase in reflection.

**Figure 4:**
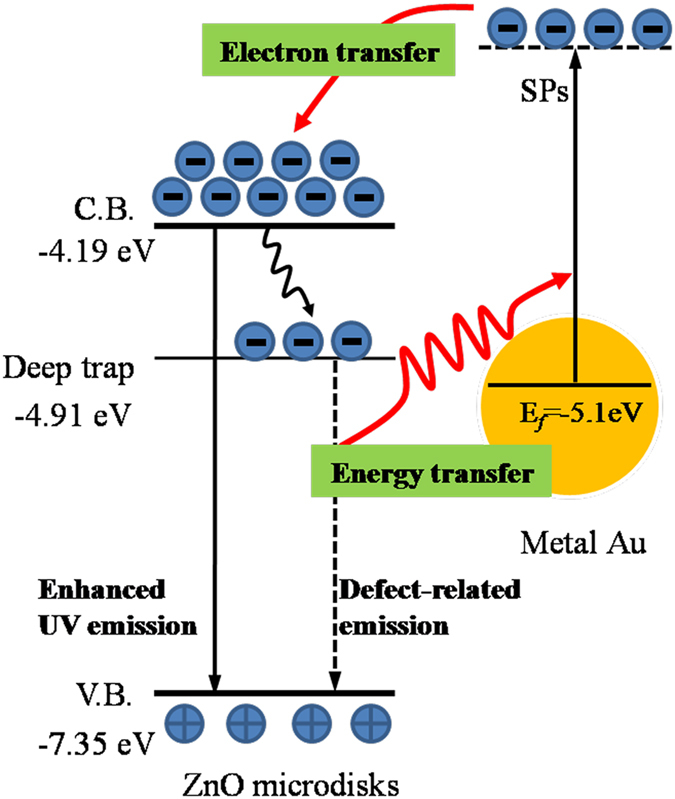
The schematic diagram of the energy coupling and plasmon-assisted electron transfer in ZnO/Au hybrid microdisks.

The defect emission of ZnO has been attributed to the transition of electrons from a deep trap level to the valence band of ZnO^3^,^5^,^18^. This emission is indeed decreased even disappeared almost after Au NPs-decoration in the present case. It is noted that the absorption spectra of the Au SPs are just overlapped with the defect-related emission band of ZnO in the visible range, which is impossible to couple directly with NBE of ZnO. In other words, a resonant energy coupling must be happened between the defect emission of ZnO and Au SP. Moreover, the Au NPs can be absorbed the emitted visible light photon energy of ZnO, which excites the electrons of Au to a higher energy state. So, the energy transfer is responsible for the suppression of the defect level emission. Assisted by the resonant energy coupling process, the electrons with higher energy can be transferred favorably to the conduction band of ZnO, thus resulting in an enhancement of NBE of ZnO, as shown the diagram in Fig. 4. Briefly, the photon energy of the defect emission of ZnO is reabsorbed resonantly by Au NPs to generate LSP, which excites the electrons of Au to a higher energy state and then transfer to the conduction band of ZnO. This plasmon-assisted electron transfer process results in the visible light suppression and the NBE enhancement of ZnO. The similar behaviors have also been reported in our previous reports^3^,^19^. A similar SP-assisted electron transfer process has also been observed in ZnO quantum dots and ZnO microflowers decorated by Au NPs and confirmed by time-resolved photoluminescence (TRPL) in our previous work^19^,^20^, which exhibits the delayed lifetime of the photoluminescence. According to the diagram, the enhancement of NBE intensity is related to several factors: (1) the overlap extents between the defect emission spectra of ZnO and absorption spectra of Au NPs, which indicates the coupling efficiency; (2) the absorbance of Au NPs, which hints the coupling strength and (3) the morphology and spatial density of the Au NPs, which influences the local field enhancement induced by hot-spots effect^21^,^22^. With increasing the sputtering time of Au NPs from 0 s to 90 s, the absorbance of Au NPs is increased gradually even in the reduction of spectral overlap. At the sputtering time of 90 s, Au NPs with denser distribution have the strongest absorption at 563 nm although there is an appearance of some deviation from the defect emission peak of as-grown ZnO microdisks at 508.12 nm. Due to the improved coupling and strongest local field enhancement, the free electrons of Au NPs can be excited efficiently to a higher energy state of Au NPs by SPs coupling and then transferred into the conduction band of ZnO. As a result, there is an occurrence of the strongest NBE in this case. When the sputtering time is increased to 105 s, the gathered Au NPs raise the reflection and scattering of the excitation light, weaken the absorption and also hinder the light emitted out, thus leading to the decrease in enhancements of coupling strength and local field.

**Figure 5:**
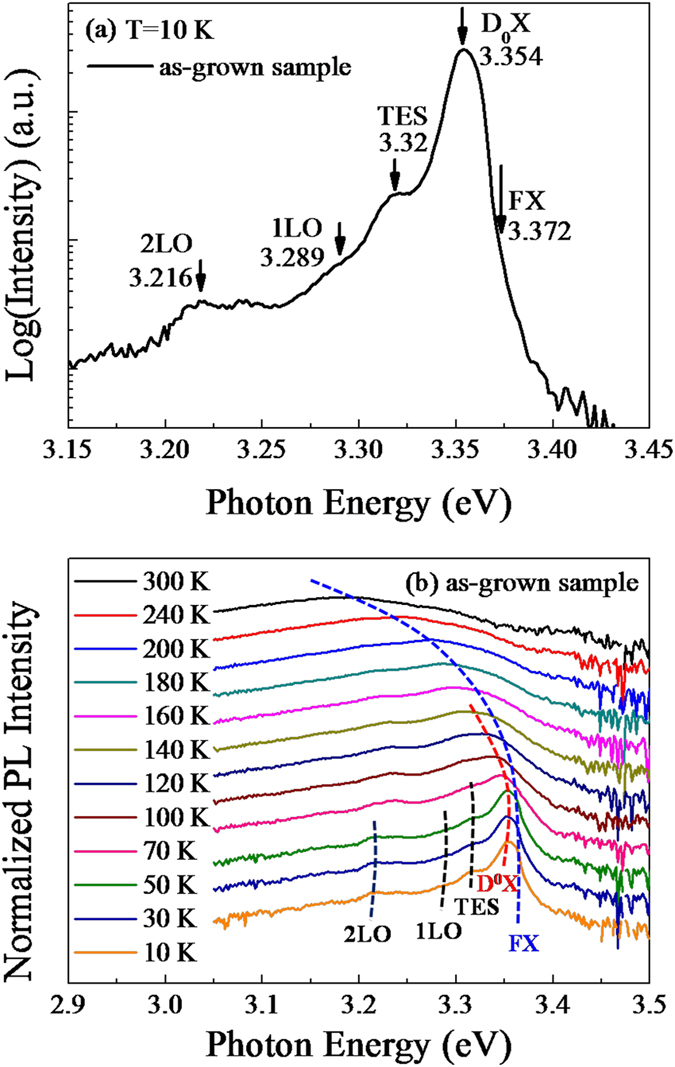
(**a**) PL spectra of as-grown ZnO microdisks at 10 K and (**b**) Temperature-dependent normalized PL spectra of the as-grown sample.

To further explore the coupling mechanism between the metal SPs and the ZnO emission, temperature-dependent PL spectra were conducted from the ZnO microdisks before and after the decoration of Au NPs. The PL spectrum of as-grown ZnO at 10 K is plotted in Fig. 5(a). It shows a dominant peak at around 3.354 eV and a weak shoulder at around 3.372 eV, which correspond to the neutral donor bound exciton emission (D^0^X) and free exciton (FX) recombination^23^,^24^. The peak with weak intensity at 3.32 eV is attributed to the two-electron satellite (TES) transition of the D^0^Xs^25^,^26^. In a lower energy side, another two weak peaks with an constant energy separation of ~73 meV from D^0^Xs are assigned as the first and the second order longitudinal optical (LO) phonon replicas of D^0^Xs^27^. Figure 5(b) represents the temperature-dependent PL spectra of the as-grown sample with temperatures ranging from 10 K to 300 K. As the temperature increases, more and more D^0^Xs are thermally activated and converted to FXs, which is one of the characteristics of normal thermal behavior. Both D^0^Xs and FXs are shifted to the longer wavelength with a presence of weaker intensity as increasing the temperature, and also the intensities of the FXs peaks with higher energy become dominant in these spectra.

**Figure 6:**
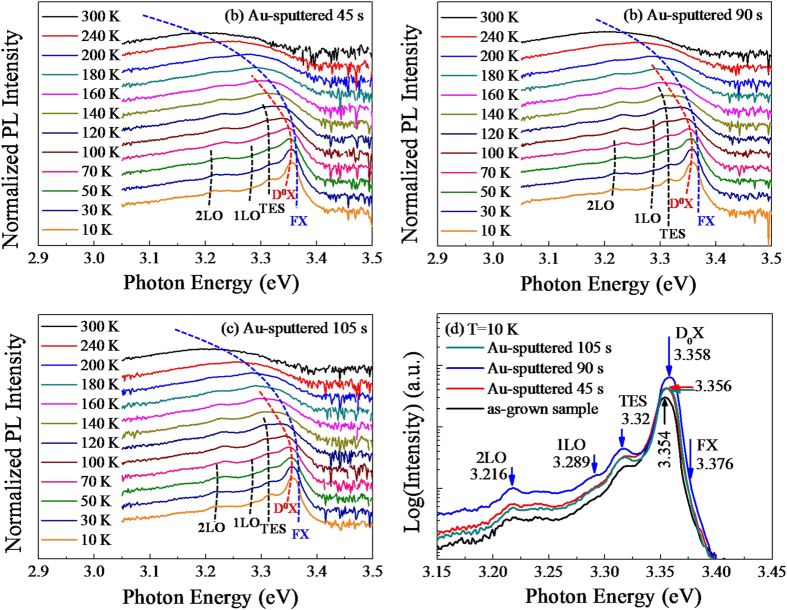
Temperature-dependent PL spectra of the Au-sputtered ZnO microdisks (**a**) 45 s, (**b**) 90 s and (**c**) 105 s. (**d**) PL spectra of as-grown sample and Au-sputtered ZnO microdisks at 10 K.

The temperature-dependent PL spectra of the Au-sputtered microdisks with the sputtering times of 45 s, 90 s and 105 s are shown in Figure 6(a,b,c), respectively. For the Au sputtered samples, the spectra similar to that of as-grown ZnO microdisks are displayed a red shift in the peak energy and there is an occurrence of decrease in intensity with the temperature increasing. D^0^Xs can be transformed gradually into FXs with increasing the temperature; consequently, the relative intensities of the FXs emission can be increased obviously and eventually became dominant in the room temperature PL spectra, which indicated by the dotted lines in Fig. 6(a–c). At 10 K, the representative PL spectra of ZnO microdisks before and after the decoration of Au NPs are compared in Fig. 6(d). The emissions at 3.356 eV, 3.358 eV and 3.356 eV are attributed to D^0^Xs for the Au-sputtered samples 45 s, 90 s and 105 s, respectively. With increasing the sputtering times of Au NPs from 0 s to 45 s and to 90 s, the D^0^Xs become stronger due to gradually improved coupling of higher radiative recombination probability in the structure of ZnO/Au, which is similar to the variation at room temperature. Then, the D^0^Xs are reduced with further increasing the sputtering time of Au NPs to 105 s because of the Au NPs merging together, thus leading to a decrease in the coupling strength. A slight blue shift of 4 meV is noted in comparison with that of as-grown ZnO sample when increasing the sputtering times of Au NPs from 0 s to 90 s. Such a spectral blue shift has been reported previously in the CdS quantum dots and NWs^28^,^29^,^30^,^31^ and could be arised from a few origins: (1) the Burstein-Moss effect due to the electrons filling to conduction band^7^,^8^; (2) an increase of excitonic energy due to excess surface-trapped electrons^32^,^33^; (3) a decrease in the oscillator strength of the excitonic transitions due to trapped elcetrons and holes^31^,^34^. Mechanisms (2) and (3) could be ruled out as these would be more dominant for small semiconductor clusters/NWs with very large surface to volume ratios (e.g., in the case where the diameter of particle/NW is comparable to the Bohr radius of ~3 nm). So, it is reasonable to attribute the blue shift of the optical band gap to the BM effect in the present case. The BM shift (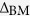) can be expressed as follows^9^:


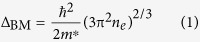


where 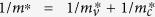, 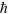 is the Planck constant, 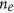 is the electron concentration, the reduced mass 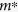 is derived from the effective mass of the electrons in the valence and conduction band with 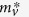
= 0.25 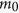 and 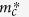
= 0.59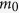 (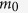
= free electron mass) respectively^35^,^36^. According to the above equation, the BM shift changed with the electron concentration. Also, according to the equation of *n*_*e *_= *β*⋅*I*_exc_/*hω*_*exc*_^37^, where the absorption coefficient *β* ≈1.6 × 10^5^ cm^−1^, the excited electron concentration was estimated as 5.088 × 10^19^ cm^−3^ under the excitation power of about 159 μJ/cm^2^ for the as-grown ZnO. After the decoration of Au NPs on the ZnO surfaces, the electron concentration in the conduction band of ZnO can be further increased due to SP-assisted electron transfer, thus presented the BM effect. The BM effect has also been observed in the similar electron concentration from other research groups^36^,^38^. Furthermore, the electron transfer can be blocked if the ZnO and Au NPs were separated by an insulating layer of SiO_2_, whereas the intensity of the samples is decreased with increasing the sputtering time of Au NPs. This also indicates the electron transfer process in the situation without any blocking layer. When the sputtering time is prolonged from 0 s to 90 s, the concentration of the free electrons filling in the conduction band of ZnO microdisks is increased gradually due to an assistance of the Au SPs, which accompanies a gradual enhancement in NBE intensity of ZnO and a blue shift of the peaks. For the 90 s sample, the contributions for the absorbance of Au SPs and coupling can be reached the maximum; the free electrons of Au NPs can be excited efficiently and then transferred into the conduction band of ZnO, thus leading to an enhancement in the BM effect. While a slight red shift of 2 meV relative to sample of 90 s but still blue shift of 2 meV relative to the as-grown sample is observed due to the Au NPs merged together as increasing the sputtering time of Au from 90 s to 105 s, giving rise to a low probability of free electron excited effectively. So the concentration of free electrons is reduced to fill the conduction band of ZnO microdisks because of its weak BM effect. In addition, it can be observed that a maximum blue shift at room temperature is 44 meV and 4 meV at 10 K. Two factors are beneficial to the electrons filling in the conduction band of ZnO and hence to the higher blue shift at higher temperature: in one hand, more electrons can be ionized to the conduction band from the shallow levels at higher temperature; in the other hand, the defect-related emission at room temperature is much stronger than that of the low temperature, which excites more surface plasmons of Au NPs, thus resulting in electrons transfer with higher density to the conduction band of ZnO.

**Figure 7:**
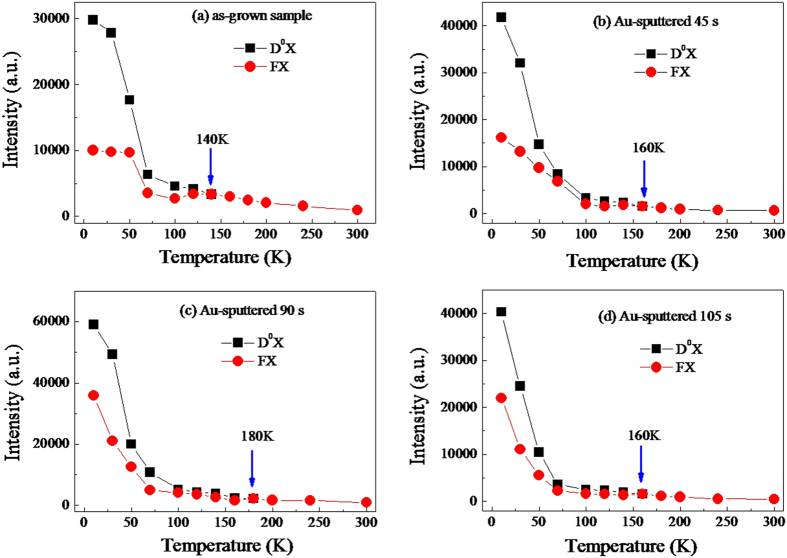
Temperature-dependent PL intensity of D^0^X and FX excitonic emissions for (**a**) as-grown sample and Au-sputtered (**b**) 45 s, (**c**) 90 s and (**d**) 105 s samples.

Figure 7 illustrates the temperature-dependent PL intensity of D^0^Xs and FXs excitonic emission as a function of temperature for the as-grown and Au-sputtered samples. As a whole, with increasing the temperature, the PL intensities of all the samples are decreased almost linearly before 100 K, whereas the Au-sputtered samples decreased more quickly, especially the Au-sputtered 90 s sample. However, the PL intensities of all the samples are reduced very slowly as increasing the temperature from 100 K to 300 K. Corresponding to the sputtering time from 0 s to 90 s and to 105 s, the PL intensities of D^0^Xs and FXs for all the samples are increased first and then decreased at 10 K, which is similar to the variation trend of room temperature. Figure 7(a) reveals that the D^0^Xs are thermally activated and then converted to FXs at 140 K for the as-grown ZnO microdisks. For the Au-decorated samples with sputtering times of 45 s, 90 s and 105 s, the conversion points are identified at 160 K, 180 K and 160 K as shown in Fig. 7(b,c,d), respectively. When the sputtering time of Au NPs is prolonged from 45 s to 90 s, the free electrons of Au NPs are increased gradually and the BM shift is enhanced relative to as-grown sample, indicating the conversion points of the 45 s to 90 s Au-sputtered samples are from 160 K to 180 K. A stronger BM effect corresponds to a higher conversion point in the temperature-dependent PL spectra. With the sputtering time increased to 105 s, the BM effect becomes weaker due to the Au NPs merging together as shown in Fig. 3(d), whereas the conversion point is turned back to 160 K. The change in conversion point is estimated as the thermal activation energy of 1.73 meV according to the thermodynamics, which is consistent well with the blue shift of 2 meV.

In summary, we have utilized SP-assisted multi-electron-transfer process to improve the NBE emission and suppress the defect-related emission in ZnO/Au hybrid microstructures. The enhancement and blue-shift in NBE emission can be attributed to the hot electrons filling in the conduction band of ZnO. In addition, the results of temperature-dependent PL further confirmed that the enhancement in exciton intensities and the spectral blue shift were caused by the SP-exciton coupling-related BM effect. Our results are helpful and meaningful in the designing of highly efficient UV optoelectronic devices without any defect-related energy loss.

## Methods

The wurtzite structured ZnO microdisks were fabricated on silicon (Si) substrate through a simple VPT process. The Si substrate (3.5 cm * 3 cm) was ultrasonically cleaned with acetone, ethanol, and deionized water for 15 min successively and then dried by N_2_. After that, 0.75 g of ZnO and 0.75 g of graphite powders were mixed evenly and filled in a ceramic boat. The ceramic boat filled with source materials was placed in a tube with a length of 20 cm and diameter of 3.5 cm. A piece of Si substrate was covered on the boat with the polished surface facing down. The tube loaded with the ceramic boat and the Si substrate was then pushed into a tubular furnace, and the temperature was maintained at 1000 °C for 40 min. During the reaction process, oxygen and argon gases were loaded in the horizontal furnace with the flow rates of 15 sccm and 150 sccm, respectively. After the reaction, the ZnO microdisks were formed on the Si substrate, which taken out from the furnace and cooled down to the room temperature naturally.

The Au NPs were sputtered on the as-grown ZnO microdisks and cleaned Si and quartz substrates by using a small ion beam sputtering machine at the same time. The absorption spectra of Au NPs on the quartz substrates were detected by the UV-visible spectrophotometer (Shimadzu UV-2600). In order to compare the optical properties of ZnO microdisks before and after the decoration of Au NPs, the whole piece of Si substrate with ZnO microdisks was cut into five pieces and then sputtered the Au NPs with different times such as 0 s, 15 s, 45 s, 90 s, and 105 s, respectively. The morphology and structure of the samples were characterized by a field emission scanning electron microscopy (FESEM, Carl Zeiss Ultra Plus) equipped with an X-ray energy dispersive spectrometer (EDS) (Oxford X-Max 50) and the X-ray diffraction (XRD-7000, Shimadzu) with Cu Kα radiation (λ = 0.15406 nm). The room temperature PL spectra of the ZnO microdisks were measured by using a fluorescence spectrophotometer (F-4600, Hitachi) with Xe lamp as source at the excitation wavelength of 325 nm. Temperature-dependent PL (10 K-300 K) experiment was performed by using a liquid-nitrogen-cooled cryostat (Janis ST-500, Microscope Cryostat) with an excitation source of femtosecond pulse laser at 325 nm. The emission signal was collected by a fiber and then coupled into a charge-coupled-device array detector in an optical-multichannel analysizer (OMA) system. All the measurements were performed at room temperature.

## Additional Information

**How to cite this article**: Zhu, Q. *et al*. Burstein-Moss Effect Behind Au Surface Plasmon Enhanced Intrinsic Emission of ZnO Microdisks. *Sci. Rep.* **6**, 36194; doi: 10.1038/srep36194 (2016).

**Publisher’s note:** Springer Nature remains neutral with regard to jurisdictional claims in published maps and institutional affiliations.
